# Digital Pathology Data Brokerage: A Standard Recommendation for Complex Digital Pathology Information Web-Services

**DOI:** 10.1155/2014/286383

**Published:** 2014-12-01

**Authors:** Aristidis Anagnostakis, Agelos Pappas, Yves Sucaet, Wim Waelput

**Affiliations:** ^1^Technological Educational Institute (TEI) of Epirus, Faculty of Finance and Management, Enanti Gefyra Arachthou, 47100 Arta, Greece; ^2^Smartcode Software Development, 3rd Km National Road Artas-Antirriou, 47100 Arta, Greece; ^3^Pathomation BVBA, Prinses J. Charlottelaan 10, 2600 Berchem, Belgium

## Abstract

A novel recommendation for the Digital Pathology Information Web-Services (DPIWS) standard is presented, with respect to specific characteristics of the informative content of discourse. The recommendation establishes a common software interface for the exchange of digital pathology (DP) images and related metadata over the web, independently of storage, encoding, and internal handling details. The proposed structure is implemented and tested in a “Pathomation” software environment.

## Background

One of the major obstacles to establishing and adopting effective telepathology processes over time has been their lack of information brokerage standardization.

Digital pathology information is characterized by a complex structure and high data volumes. Effective handling and sharing demands in-depth interdisciplinary skills, which, along with industrial vendor proprietary formatting and data locking, makes essential information brokerage a challenging process.

Digital pathology imagery and annotation distribution is, to date, partially covered by specific portions of Digital Imaging and Communications in Medicine (DICOM) and Open Microscopy Environment (OME) standards. In addition, DP information sharing bears significant similarities to other disciplines (e.g., Geographical Information Systems), the distribution of which has been highly standardized for decades.

The proposed recommendation (DPIWS) delivers a standard web interface definition allowing requests for DP information elements handling and sharing across the web, through platform-independent and image format-agnostic calls.

## Methods

DPIWS comes as an independent recommendation. It strictly conforms to and expands the DICOM standard, with respect to the Open Microscopy Environment, and the Open Geospatial Consortium Web Map Service (WMS) and Web Feature Service (WFS) standard.

Digital pathology images are partially covered by the DICOM standard, according to the procedures of the DICOM Standards Committee 2011, Part 3: Information Object Definitions under VL Microscopic Image Information Object Definition Content Constraints A.32.2 and VL Whole Slide Microscopy IOD Content Constraints A32.8. In addition, generic URL requests for retrieving a DICOM Visual Light Image are defined under DICOM 2011, Part 18: Web Access to DICOM Persistent Objects (WADO). However, the response is a single, standard encoding image with all annotations rendered (burned) on the image; no method for requesting or handling annotations in any form other than an image is identified.

Metadata, like annotations, on microscopy images are, on the other hand, exhaustively covered by the Open Microscopy Environment: Model and Formats 2013-06 Documentation in a well-structured manner. 2-D and 3-D regions of interest (RoI) may be defined and treated and a series of annotation elements identified and supported; this makes OME an appropriate tool for handling DP annotations.

In addition, the Open Geospatial Consortium (OGC), through Web Map and Web Feature Services (WFS), effectively defines the web-handling of multiple-layered, complex raster images and vector data, making it the ideal interface to (a) uniformly query and retrieve composite large-scale DP images consisting of superimposed multiresolution raster vector and textual annotation layers and (b) uniformly share the results across the web, despite the complexity and structure of the requested content.

## Results

DPIWS is an interdisciplinary standard, designed to address the specificities of DP information brokerage needs.

For this, it (a) conforms to DICOM: images treated internally and served by DICOM-compliant systems may distribute content to DPIWS compliant clients, as is; (b) adopts the OME annotation structure to form annotation-handling requests based on well-structured XML OME annotations; and (c) adopts and extends the WFS interface for information web-handling and delivery operations.

Building DPIWS-compliant services will eventually eliminate the risk of long-term vendor content locking, thereby boosting the digital transition in the field of pathology.

## Conclusions

A standard for DP information brokerage across the web is defined, implemented, and tested in a real-life, cloud-based, distributed operating environment. The standard effectively manages the specificity of DP information by leveraging well-established interdisciplinary methodologies.

